# Effects of Anesthesia with Pentobarbital/Ketamine on Mitochondrial Permeability Transition Pore Opening and Ischemic Brain Damage

**DOI:** 10.3390/biomedicines12102342

**Published:** 2024-10-15

**Authors:** Evelina Rekuviene, Laima Ivanoviene, Vilmante Borutaite, Ramune Morkuniene

**Affiliations:** 1 Neuroscience Institute, Lithuanian University of Health Sciences, Eiveniu 4, LT-50161 Kaunas, Lithuania; vilmante.borutaite@lsmu.lt (V.B.); ramune.morkuniene@lsmu.lt (R.M.); 2 Department of Biochemistry, Lithuanian University of Health Sciences, Eiveniu 4, LT-50161 Kaunas, Lithuania; laima.ivanoviene@lsmu.lt; 3 Department of Drug Chemistry, Lithuanian University of Health Sciences, Sukileliu 13, LT-50162 Kaunas, Lithuania

**Keywords:** anesthesia, sodium pentobarbital, brain ischemia, complex I, mitochondrial permeability transition

## Abstract

Background and Objective: The alteration of mitochondrial functions, especially the opening of the mitochondrial permeability transition pore (mPTP), has been proposed as a key mechanism in the development of lesions in cerebral ischemia, wherefore it is considered as an important target for drugs against ischemic injury. In this study, we aimed to investigate the effects of mitochondrial complex I inhibitors as possible regulators of mPTP using an in vitro brain ischemia model of the pentobarbital/ketamine (PBK)-anesthetized rats. Results: We found that PBK anesthesia itself delayed Ca^2+^-induced mPTP opening and partially recovered the respiratory functions of mitochondria, isolated from rat brain cortex and cerebellum. In addition, PBK reduced cell death in rat brain slices of cerebral cortex and cerebellum. PBK inhibited the adenosine diphosphate (ADP)-stimulated respiration of isolated cortical and cerebellar mitochondria respiring with complex I-dependent substrates pyruvate and malate. Moreover, pentobarbital alone directly increased the resistance of isolated cortex mitochondria to Ca^2+^-induced activation of mPTP and inhibited complex I-dependent respiration and mitochondrial complex I activity. In contrast, ketamine had no direct effect on functions of isolated normal cortex and cerebellum mitochondria. Conclusions: Altogether, this suggests that modulation of mitochondrial complex I activity by pentobarbital during PBK anesthesia may increase the resistance of mitochondria to mPTP opening, which is considered the key event in brain cell necrosis during ischemia.

## 1. Introduction

Ischemic stroke is one of the most common causes of human death and is linked to many pathologies, such as atherosclerosis, cerebral vasoconstriction, and heart defects. The large number of neurons die during ischemic stroke; therefore, the search for clinically effective neuroprotection during brain ischemia is under intensive investigation. Opening of the mitochondrial permeability transition pore (mPTP) is considered to be one of the important events during cerebral ischemia leading to cell death [1,2,3,4]; therefore, regulation of mPTP and improvement in mitochondrial functions might be potential targets for a disease-modifying treatment strategy in brain ischemia [5,6]. It was previously established that different brain subregions showed different sensitivities to Ca^2+^-induced mPTP [7,8] and that the greatest susceptibility to mPTP was found in neuronal mitochondria [9]. The human brain has a different ratio of neurons to glia cells, far more glia in the cerebral cortex, and a high density of neurons in cerebellum [10]; therefore, the susceptibility of brain subregions to ischemia may be associated with different mitochondrial responses to mPTP activation, ultimately resulting in neuronal cell death. However, how mPTP opening is regulated is still under discussion [11].

The mPTP can be specifically blocked by cyclosporin A (CsA) during cerebral ischemia [12,13,14], yet the applicability of CsA in clinical practice is limited by side effects and the low uptake of CsA to the brain [15,16,17]. On the other hand, experimental evidence suggests that inhibition of mitochondrial respiratory chain complex I by rotenone may play an important role in mPTP regulation in several cell lines, and liver and heart mitochondria [18,19]. In addition, we and other researchers have found that rotenone reduced hypoxia/ischemia-triggered mPTP opening, mitochondrial H_2_O_2_ emission, and cerebral infarct volume in animal models [20,21]. Furthermore, growing evidence indicates that some anti-diabetic drugs (metformin, phenformin, and imeglimin) that also inhibit complex I acutely prevented mPTP opening and were neuroprotective against ischemic brain injury [22,23,24]. Previous studies have demonstrated the beneficial effects of barbiturates in hypoxic/ischemic injury. Although the effect of barbiturates, known as reversible inhibitors of complex I [25,26], has been associated with decreased consumption of cerebral oxygen and glucose [27,28], or decreased lipid peroxidation in neonates with hypoxic ischemic encephalopathy [29], these studies do not sufficiently explain neuroprotective mechanisms of barbiturates in ischemic brain cells.

Currently, phenobarbital is the first-line drug for the treatment of neonatal seizures [30,31,32]. Pentobarbital, as one of the oldest medicines in clinical use, has faster brain penetration and is used for the treatment of seizures, status epilepticus, and short-term insomnia [33]. In addition, pentobarbital is commonly used to anesthetize animals; however, it might cause severe damage to the cardiovascular system [34]. Therefore, combined pentobarbital and ketamine anesthesia (PBK) is recommended in animal ischemia/reperfusion models [34]. In this study, we aimed to investigate the effects of PBK on mPTP, mitochondrial respiration function, and cell death in the rat brain cortex and cerebellum after ischemia and whether these effects are related with mitochondrial complex I inhibition.

## 2. Materials and Methods

### 2.1. Materials

Calcium Green-5N, Amplex Red and propidium iodide were purchased from Molecular Probes/Invitrogen (by Thermo Fisher Scientific, Waltham, MA, USA); HBBS was from GIBCO (by Thermo Fisher Scientific, Waltham, MA, USA). All other reagents were purchased from Sigma-Aldrich (Steinheim, Germany).

### 2.2. Animals and Experimental Protocols of Induction of Anesthesia and Global Brain Ischemia

All experimental procedures were reviewed according to the European Convention for the protection of vertebrate animals used for experimental and other scientific purposes, as well as Directive 2010/63/EU with respect to the Republic of Lithuania law on the care, housing, and utilization of animals and approved by the National Ethical Committee for Animal Care (2019-04-23 No. 62-106). The rats were bred and maintained at Lithuanian University of Health Sciences Animal House under controlled conditions.

Studies were performed with respiratory chain complex I inhibitor pentobarbital, evaluating (1) the effect of PBK infusions over 2 h ischemia on the mitochondrial respiration functions of different brain areas (cortex, cerebellum), mPTP activation, reactive oxygen species (ROS), respiratory chain complex I activity, and brain cell viability in brain slices; (2) the direct effect of pentobarbital on the mPTP opening and respiration functions of isolated rat brain (cortex and cerebellum) mitochondria.

Wistar male rats (8–12 weeks) were randomly divided into four experimental groups. Control group (I): rats were anesthetized with carbon dioxide (98%) at room temperature and then killed by decapitation. Brains were removed to isolate mitochondria and prepare brain slice cultures. For the 120 min ischemia group (II): animals were anesthetized with carbon dioxide, then brains were placed in a hypoxia chamber (93% N_2_, 5% CO_2_, 2% O_2_; 37 °C) for 2 h. PBK control group (III): rats were given anesthesia with a combination of ketamine (100 mg/kg injected intramuscularly (IM)) plus sodium pentobarbital (40 mg/kg, intraperitoneally (IP)) for 20 min as described in [34]. Then, the brains were used for isolation of mitochondria and preparation of brain slices. PBK plus 120 min ischemia group (IV): animals received PBK as above, and then their brains were placed in a hypoxic chamber for 2 h. After the indicated procedures, brain cortex and cerebellum were cut separately. The isolation medium composition was 225 mM mannitol, 75 mM sucrose, 5 mM HEPES, 1 mM EGTA, pH 7.4 at 4 °C.

### 2.3. Isolation of Mitochondria from Cortex and Cerebellum

All procedures for isolating mitochondria were performed on ice to maintain the temperature of 4 °C. Selected brain regions, cortex and cerebellum, were placed in isolation media and homogenize separately using a glass–Teflon homogenizer. Samples were spun at 1000× *g* for 5 min and 10,000× *g* for 10 min (Thermo Scientific Biofuge Stratos, USA/Canada). The mitochondria were then suspended in isolation medium, and total mitochondrial protein was determined by a modified Biuret method [35].

### 2.4. Measurement of Mitochondrial Calcium Retention Capacity (CRC)

Calcium retention capacity (CRC) of brain mitochondria was determined fluorometrically (Fluorescence Spectrometer Perkin Elmer LS55, Shelton, CT, USA) using fluorescent dye Calcium Green-5N 100 nM (excitation at 507 nm, emission at 536 nm) in medium containing 200 mM sucrose, 10 mM Tris-HCl, 1 mM KH_2_PO_4_, 10 μM ethylene glycol tetraacetic acid (EGTA), and 5 mM succinate, pH 7.4 at 37°C, by measuring the external Ca^2+^ concentration that induces Ca^2+^ release through the mPTP. Experiments were initiated by adding 0.2 mg/mL mitochondria to the cuvette, followed by additions of 1.67 µM CaCl_2_ (at approximately 2 min intervals), which increased extramitochondrial calcium ion fluorescence. As the mitochondria took up calcium, the fluorescence signal decreased. Calcium ions were continuously added until a large increase in fluorescence was observed, indicating that the mPTP opened to release the stored Ca^2+^. In experiments on the direct effect of sodium pentobarbital or ketamine on CRC, isolated control mitochondria were incubated for 2 min in the absence/presence of various concentrations of pentobarbital (0.4–2 mM) or ketamine (0.4–1 mM); then, pulses of Ca^2+^ (1.67 µM) were added until the opening of the mPTP. The signal was calibrated by adding a known amount of CaCl_2_. Total mitochondrial Ca^2+^ retention capacity is expressed as nmol/mg mitochondrial protein.

### 2.5. Measurement of Mitochondrial Respiratory Functions

Mitochondrial oxygen consumption was measured oxygraphically with an Oroboros instrument (Oroboros Instruments, Insbruck, Austria) at 37 °C in 2 mL respiration buffer containing 110 mM KCl, 10 mM Tris-HCl, 5 mM KH_2_PO_4_, 2.24 mM MgCl_2_, pH 7.2, and 0.25 mg/mL mitochondrial protein. Mitochondrial proton LEAK respiration rate (V_L_) was measured using substrates pyruvate (1 mM) plus malate (1 mM) in the absence of adenosine diphosphate (ADP) as non-phosphorylating resting state. Mitochondrial ADP-stimulated respiration rate (V_ADP_) was achieved by adding 2 mM ADP with substrates pyruvate (1 mM) and malate (1 mM) or succinate (5 mM) plus amytal (1.5 mM). V_Atr_ was registered for succinate plus amytal oxidation with atractyloside (100 μM). The mitochondrial respiration was expressed as pmol O_2_/s/mg of mitochondrial protein.

### 2.6. Measurement of Complex I Activity

Mitochondrial complex I activity was determined as the rate of reduced nicotinamide adenine dinucleotide (NADH) oxidation at 37 °C in the reaction mixture, which contained: 100 µM NADH, 25 mM KH_2_PO_4_, 60 µM coenzyme Q_1_, 2 µg/mL antimycin, 2 mM sodium azide, 3 mg/mL bovine serum albumin (BSA), and 0.125 mg/mL frozen–thawed/sonicated mitochondria (pH 7.4). Complex I activity was determined as the difference in NADH oxidation rate in the absence and presence of 10 μM rotenone and was expressed as NADH nmol/min/mg mitochondrial protein.

### 2.7. Measurement of Mitochondrial H_2_O_2_ Generation

H_2_O_2_ generation by isolated mitochondria was determined fluorometrically with a Thermo Scientific Plate reader Fluoroskan Ascent (Vantaa, Finland) in 200 µL of respiration buffer (see composition in Section 2.5) and substrates 1 mM pyruvate plus 1 mM malate or 5 mM succinate at 25 °C, with 0.05 mg/mL mitochondria, 10 μM Amplex Red, and 5 U/mL horseradish peroxidase Type IV-A (excitation at 544 nm, emission at 590 nm). The fluorescence signal was calibrated using a known H_2_O_2_ concentration.

### 2.8. Preparation of Rat Brain Tissue Slices and Evaluation of Cell Death

Rat brains were immersed in ice cold HBBS buffer with 13 mM glucose. For experiments with 2,3,5-triphenyltetrazolium chloride (TTC), tissue coronal sections (slices) of cerebral cortex and cerebellum were prepared with a vibratome (Vibratome 1000, Technical Products International Inc., West Bend, WI, USA), thickness 1 mm. Following preparation, slices were stained with 2% TTC solution, keeping the cuts for 15 min in the dark at 37 °C, followed by 15 min with 4% buffered formalin solution. Stained and fixed sections were photographed with a Nikon D3100 digital camera (Tokyo, Japan). Images of brain sections were analyzed with the ImageJ software (1.47). The area of infarction in the slices is expressed as a percentage by comparing the ratio of the whole coronal section to the damaged area (light color of the tissue).

The degree of cell death in thin brain sections was assessed by fluorescence microscopy with propidium iodide (PI) and Hoechst dyes using an OLYMPUS IX71S1F-3 fluorescence microscope (by Thermo Fisher Scientific, Waltham, MA, USA), x20 objective. The brain tissues, cortex and cerebellum, were sliced into 100 μm thick sections using a vibratome filled with HBSS and 13 mM glucose (4 °C). Sections were then transferred into 1 mL HBSS with 7 µM PI and 4 µg/mL Hoechst 33342 for 10 min and gently covered with glass coverslips. Stained sections were photographed in at least 5 randomly selected fields per slice (5–10 slices per group of individual experiment). Data were obtained from 3–5 individual experiments. Images were analyzed by Image J program (v.1.47) and expressed as the mean level of red fluorescence.

### 2.9. Statistical Analysis

IBM SPSS Statistics 20 software was used for statistical analysis. Results are expressed as mean ± SE of at least three separate experiments. Statistical analysis was performed using Analysis of Variance (ANOVA) followed by Tukey or Fisher’s Least Significant Difference (LSD) post-hoc tests. A value of *p* < 0.05 was considered as statistically significant result.

## 3. Results

### 3.1. Pentobarbital/Ketamine Anesthesia Increases Resistance of Rat Brain Cortex and Cerebellum Mitochondria to Ca^2+^-Induced Opening of mPTP during Ischemia

**Figure 1:**
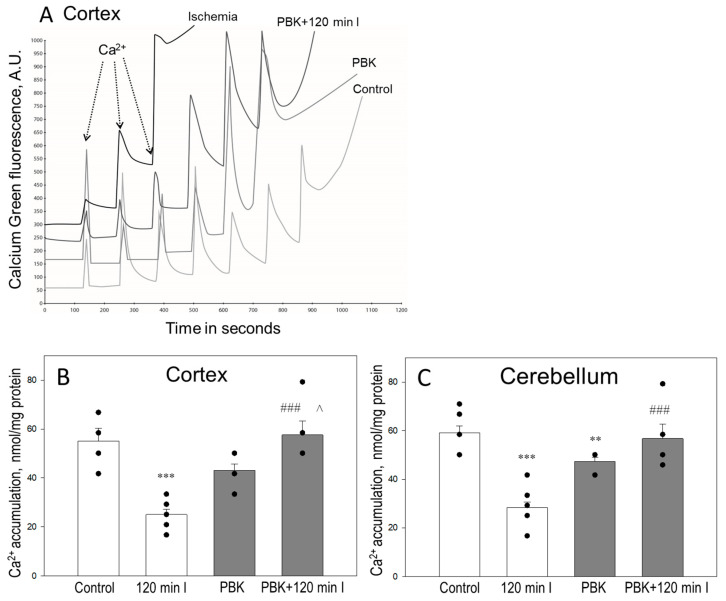
Anesthesia with pentobarbital/ketamine increases Ca^2+^ retention capacity (CRC) of ischemia-damaged mitochondria isolated from rat brain cortex and cerebellum. (**A**)—representative curves of the measurement of CRC of isolated cortex mitochondria after 120 min global brain ischemia in vitro plus/minus pentobarbital/ketamine (PBK) anesthesia. CRC of mitochondria isolated from brain cortex (**B**) and cerebellum (**C**) was measured fluorometrically using succinate as a substrate as described in Materials and Methods. **—statistically significant effect compared to control, *p* < 0.01; ***—statistically significant effect compared to control, *p* < 0.001; ^###^—statistically significant effect compared to 120 min ischemia group, *p* < 0.001; ^—statistically significant effect compared to PBK control group, *p* < 0.05. Means ± standard errors (SE) of 3–10 separate experiments are presented.

We aimed to investigate whether the application of pentobarbital/ketamine (PBK) can increase the capability of cortex and cerebellum mitochondria to retain calcium during ischemia. Isolated mitochondria of all groups were energized with succinate and then were treated with pulses of Ca^2+^ (1.67 µM) until the opening of the mPTP (representation of the curves shown in the Figure 1A). The assay used measures the sensitivity of mitochondria to Ca^2+^, which is referred to as Ca^2+^ retention capacity (CRC). The quantitative analysis of results showed that control mitochondria of the brain cortex were capable to accumulate Ca^2+^ to a level of 55 ± 5 nmol/mg protein (Figure 1B). One hundred and twenty minutes of ischemia markedly reduced the CRC of cortex mitochondria to 32 ± 1 nmol/mg protein. PBK control of cortex mitochondria showed similar CRC as control, 45 ± 3 nmol/mg protein. However, the CRC of cortex mitochondria isolated after PBK anesthesia and 120 min ischemia was similar to that of the control and slightly higher than in the PBK control group: 57 ± 6 nmol/mg protein (Figure 1B). Similar results were obtained with cerebellum mitochondria (Figure 1C): mitochondria of the control group were able to accumulate 59 ± 3 nmol/mg protein of Ca^2+^; 120 min ischemia reduced it to 36 ± 3 nmol/mg protein; CRC of the PBK control group was 47 ± 3 nmol/mg protein; and of the PBK + 120 min group was 57 ± 6 nmol/mg protein. These results indicate that anesthesia with PBK desensitizes ischemia-damaged cortical and cerebellar mitochondria to Ca^2+^ and initiation of mPTP opening. However, there were no differences in CRC of mitochondria isolated from the cortex and cerebellum under control and ischemic conditions plus/minus PBK anesthesia.

### 3.2. Pentobarbital/Ketamine Anesthesia Prevents Respiratory Impairment in the Cortex and Cerebellum Mitochondria during Ischemia

**Figure 2:**
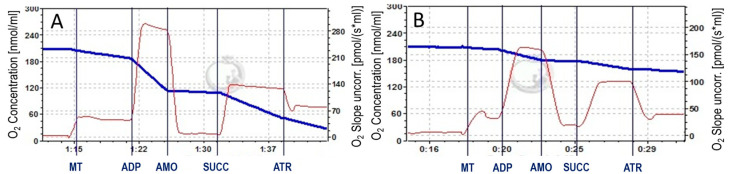

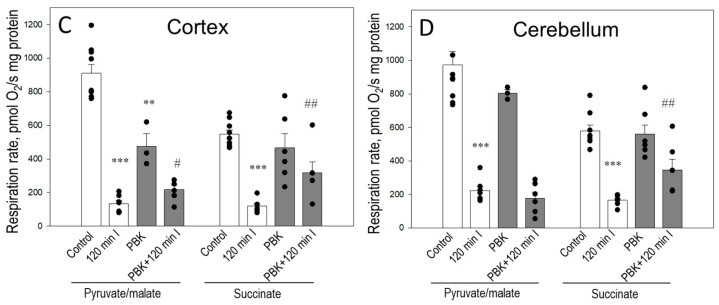
Anesthesia with pentobarbital/ketamine prevents ischemia-induced inhibition of respiration of rat brain cortex and cerebellum mitochondria. Representative original traces of respirometry measurements in cortex mitochondria, control (**A**) and PBK control (**B**); with additions: MT—mitochondria (0.25 mg protein/mL) in the presence of 1 mM pyruvate plus 1 mM malate; ADP—(2 mM); AMO—1.5 mM amobarbital; SUCC—5 mM succinate; ATR—100 μM atractyloside. Blue line indicates oxygen (O_2_) concentration in chamber; red line—O_2_ flux per chamber volume. Mitochondrial ADP-stimulated respiratory rate (V_ADP_) of cortex (**C**) and cerebellum (**D**) with pyruvate/malate (1 mM/1 mM) and 5 mM succinate plus 1.5 mM amytal were measured (see Materials and Methods). **—statistically significant effect compared to control, *p* < 0.01; ***—statistically significant effect compared to control, *p* < 0.001; ^#^—statistically significant effect compared to 120 min ischemia group, *p* < 0.05; ^##^—statistically significant effect compared to 120 min ischemia group, *p* < 0.01, #—Means ± SE of 3–9 separate experiments are presented.

Next, we measured the respiration rate of control and ischemia-damaged mitochondria isolated from rat brain cortex and cerebellum with/without PBK respiring with pyruvate/malate and succinate. As shown in Figure 2, ischemia significantly decreased V_ADP_ of isolated cortex mitochondria with pyruvate/malate and succinate (by 85% and 78%, respectively) compared to control (Figure 2A,C). Cortical mitochondria of the PBK control group exhibited partially inhibited V_ADP_ respiration with pyruvate/malate (by 48%) compared to the control, while with succinate the respiration rate was as high as in the control group (Figure 2A–C). The V_ADP_ of cortical mitochondria isolated after PBK and 120 min ischemia was significantly higher (nearly two and a half times) oxidizing succinate, though not fully recovered, and by 65% higher oxidizing pyruvate/malate compared to the corresponding 120 min ischemic group (Figure 2C).

Similar results were obtained with cerebellar mitochondria (Figure 2D): 120 min ischemia decreased V_ADP_ respiration by 77% with pyruvate/malate and by 72% with succinate compared to the corresponding control. V_ADP_ of cerebellum PBK control was not different from control with pyruvate/malate and succinate. However, V_ADP_ of the PBK + 120 min ischemia group was the same as that of 120 min ischemia group with pyruvate/malate. In contrast, V_ADP_ of PBK + 120 min ischemia was twice higher compared with 120 min ischemia group of cerebellar mitochondria respiring on succinate (Figure 2D).

Pyruvate/malate-driven V_L_ of the cortical control mitochondria was 197.5 ± 11.2 and cerebellum—185.1 ± 9.9 pmol O_2_/s/mg protein. As expected, 120 min ischemia caused a significant decrease of both types of mitochondria V_L_ with pyruvate/malate by 60% in cortex (78.6 ± 4.7 pmol O_2_/s/mg protein) and by 39% in cerebellum (113.6 ± 8.3 pmol O_2_/s/mg protein). The PBK group showed partial inhibition of pyruvate/malate-V_L_ in cortex by 41% (115.8 ± 15.9 pmol O_2_/s/mg protein) and in cerebellum by 23% (142.4 ± 9.9 pmol O_2_/s/mg protein) compared to control. However, the PBK + 120 min I group showed increase of V_L_ only in the ischemic cortical mitochondria by 81% (to 142.6 ± 18.5 pmol O_2_/s/mg protein) and remained unchanged in the cerebellum (107.6 ± 24.9 pmol O_2_/s/mg protein).

These results indicate that PBK prevented ischemia-induced suppression of ADP-stimulated respiration with succinate of both cortex and cerebellum mitochondria; however, the V_L_ and V_ADP_ with pyruvate/malate was partially preserved in brain cortex mitochondria only. Findings that PBK partially inhibits V_L_ and V_ADP_ respiration with pyruvate/malate but not succinate in the cortex control group mitochondria suggest that PBK may affect complex I activity.

### 3.3. Effect of Anesthesia with Pentobarbital/Ketamine on Complex I Activity of Rat Brain Cortex and Cerebellum Mitochondria during Ischemia

**Figure 3:**
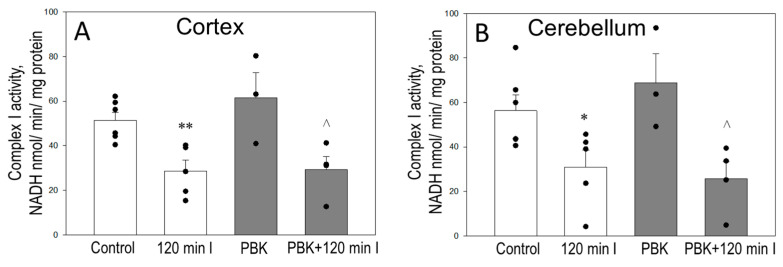
Effect of pentobarbital/ketamine anesthesia on complex I activity of cortex (**A**) and cerebellum (**B**) mitochondria during brain ischemia. Complex I activity was determined as the rate of NADH oxidation in frozen–thawed/sonicated cortex (**A**) and cerebellum (**B**) mitochondria as described in Materials and Methods. *—statistically significant effect compared to control, *p* < 0.05; **—statistically significant effect compared to control, *p* < 0.01, ^^^—statistically significant effect compared to PBK control group, *p* < 0.05. Means ± standard errors of 3–6 separate experiments are presented.

Next, we tested whether PBK can inhibit complex I activity in control and ischemia-damaged isolated, frozen–thawed/sonicated cortical and cerebellar mitochondria, measured as the NADH oxidation in the presence of coenzyme Q_1_. As expected, 120 min ischemia decreased complex I activity by 45% in isolated cortex (Figure 3A) and cerebellum (Figure 3B) mitochondria, compared with the respective control. PBK anesthesia itself did not change the activity of complex I in either cortex or cerebellum mitochondria or control groups and was as high as in the control groups, indicating that the effects of anesthesia might be rapidly reversible. However, PBK ± 120 min ischemia resulted the loss of complex I activity at a similar level to that in 120 min I group: by 45% in cortex mitochondria (Figure 3A) and by 55% in cerebellum mitochondria (Figure 3B). The results indicate that 120 min of ischemia leads to a significant decrease in the activity of complex I in both the cortex and cerebellum, which was not modified by PBK.

### 3.4. Effect of Pentobarbital/Ketamine Anesthesia on ROS Level of Rat Brain Cortex and Cerebellum Mitochondria during Ischemia

Blockade of ROS by amobarbital perfusion has been established to protect against ischemia-induced damage in heart cells [36,37]. In our study, we found that mitochondrial ROS generation assessed by H_2_O_2_ production was not significantly modified by 120 min ischemia, nor by PBK anesthesia treatment and ischemia using pyruvate/malate and succinate (Table 1).

### 3.5. Effects of Pentobarbital/Ketamine on the Level of Ischemia-Induced Necrosis in Brain Slices

**Figure 4:**
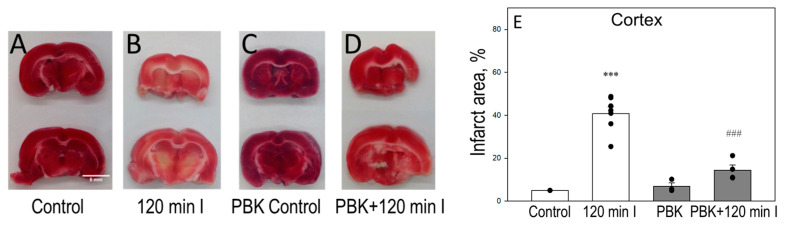
Effect of pentobarbital/ketamine anesthesia on TTC staining during brain ischemia in cortex slices. Representative images of 2,3,5-triphenyltetrazolium chloride (TTC)-stained brain cortex slices: (**A**) control; (**B**) after 120 min ischemia; (**C**) PBK control; (**D**) PBK anesthesia + 120 min ischemia. (**E**) Quantitative analysis of infarct area in each group. TTC staining was determined as described in Materials and Methods. The unstained/white intensity (infarct area) was measured by the Image J program and expressed as the percent of the whole cortex coronal section. ***—statistically significant effect, if compared to control, *p* < 0.001; ^###^—statistically significant effect, if compared to 120 min ischemia group, *p* < 0.001. Means ± SE of 3–6 separate experiments are presented.

To investigate the role of PBK on brain cell necrosis level during ischemia, we evaluated 2,3,5-triphenyltetrazolium chloride (TTC) and propidium iodide (PI) staining in cortex and cerebellum slices. For TTC measurements, control or 120 min ischemia plus/minus PBK-exposed brains, cortex and cerebellum, were sliced into 1 mm thick coronal slices and stained with TTC dye, used to label non-injured, viable tissue in red, while the infarct area remained white. In cortex slices, 120 min ischemia reduced red intensity, thus increasing the unstained infarct area by 40 ± 3% (Figure 4A,B,E). When the cerebral cortex was sliced after PBK plus ischemia, the infarction area was significantly decreased, to 16 ± 5% (Figure 4D,E). PBK control showed 8 ± 2% of infarction and was not statistically different from the control (Figure 4A,C,E).

**Figure 5:**
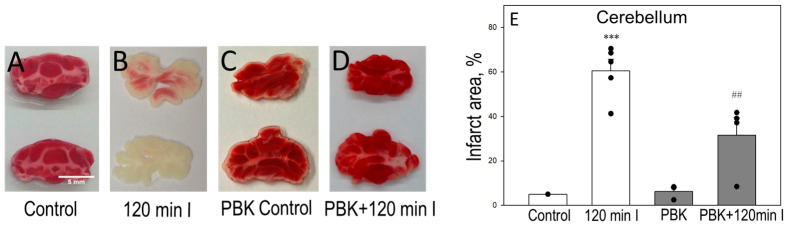
Effect of PBK on TTC staining during brain ischemia in cerebellum slices. Representative images of TTC-stained cerebellum slices: (**A**) Control; (**B**) after 120 min ischemia; (**C**) PBK control; (**D**) PBK anesthesia + 120 min ischemia (**E**) quantitative analysis of infarct area in each group. Experimental conditions were the same as described in Figure 4. ***—statistically significant effect, if compared to control, *p* < 0.001, ^##^—statistically significant effect, if compared to 120 min ischemia group, *p* < 0.01. Means ± SE of 3–6 separate experiments are presented.

In parallel, the images of cerebellum slices stained with TTC is represented in Figure 5; 120 min ischemia increased the infarct area to 60 ± 5%, whereas the exposure to PBK before the onset of ischemia resulted in 39 ± 1% infarct area (Figure 5D,E). The infarct area of PBK control was just 5 ± 3% (Figure 5C,E). These results indicate that PBK may be protective against ischemia-induced cell death in the brain cortex and cerebellum; however, the cerebellum was found more affected after 120 min ischemia with/without PBK, compared to cortex necrosis level assessed as TTC staining.

**Figure 6:**
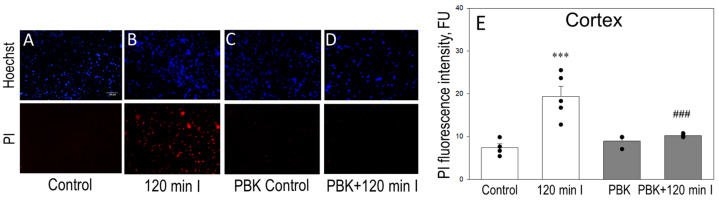
Effect of PBK on Hoechst/PI staining after brain ischemia in cortex slices. Representative images of Hoechst/propidium iodide (PI) staining of the same microscopic field in brain coronal cortex slices: (**A**) control; (**B**) after 120 min ischemia; (**C**) PBK control; (**D**) PBK anesthesia + 120 min ischemia. (**E**) Quantitative analysis of PI fluorescence intensity in each group. Hoechst/PI staining was performed in 100 µm thick coronal slices, as described in Materials and Methods. Images were analyzed by Image J program and expressed as the intensity of red fluorescence (fluorescence units, FU). ***—statistically significant effect compared to control, *p* < 0.001; ^###^—statistically significant effect compared to 120 min ischemia group, *p* < 0.01. Means ± SE of 3–5 separate experiments are presented.

We also evaluated the necrosis level after PBK anesthesia ± ischemia by PI + Hoechst staining in 100-μm-thick cortex and cerebellum slices. Figure 6 represents cortex images (upper) of Hoechst fluorescence in parallel with PI fluorescence (bottom images) of the same microscopic field. The cell damage within each slice was evaluated by the average intensities of PI (red) fluorescence and is shown in Figure 6E. The control cortex slices showed only weak PI fluorescence (6.67 FU, Figure 6A,E), while 120 min ischemia increased of PI fluorescence to 20.21 FU (Figure 6B,E). Importantly, PBK anesthesia exerted the effect on PI staining in ischemia-affected cortex and decreased red fluorescence to 10.26 FU (Figure 6D,E). PBK control showed 8.53 FU intensity and was not different of control (Figure 6C,E).

**Figure 7:**
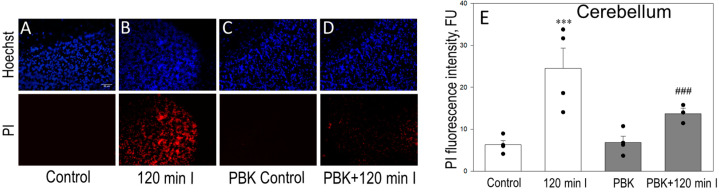
Effect of PBK on Hoechst/PI staining after brain ischemia in cerebellum slices. Representative images of Hoechst/PI staining of the same microscopic field in cerebellum slices: (**A**) control; (**B**) after 120 min ischemia; (**C**) PBK control; (**D**) PBK anesthesia + 120 min ischemia. (**E**) Quantitative analysis of PI fluorescence intensity in each group. Experimental conditions were the same as in Figure 6. ***—statistically significant effect, if compared to control, *p* < 0.001; ^###^—if compared to 120 min ischemia group, *p* < 0.01. Means ± SE of 3–5 separate experiments are presented.

Alongside, Figure 7A–D represents images of cerebellum Hoechst (upper images) and PI (bottom) fluorescence of the same microscopic field and Figure 7E represents PI (red) fluorescence intensity units, which in control were 6.72 after 120 min of ischemia—24.55; PBK + 120 min ischemia—13.34; PBK control was only 6.08 PI FU.

Altogether, these data suggest that anesthesia with pentobarbital/ketamine decreases necrosis level after global brain ischemia injury in cortex and cerebellum regions measured as TTC and PI staining with more effective protection in cortex tissue.

### 3.6. Direct Effects of Pentobarbital and Ketamine on CRC, Mitochondrial Respiration, and Complex I Activity in Isolated Rat Brain Cortex and Cerebellum Mitochondria

**Figure 8:**
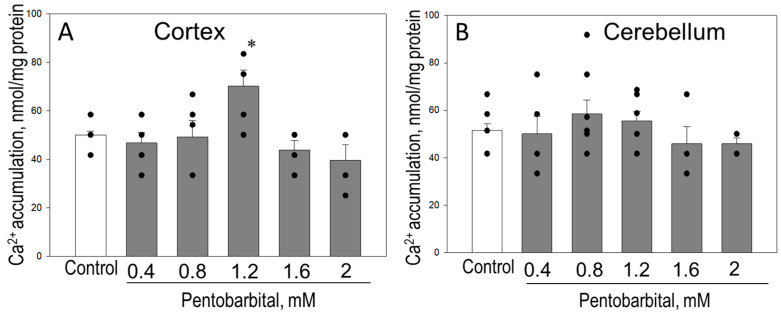
Direct effect of pentobarbital on Ca^2+^ retention capacity of isolated brain cortex and cerebellum mitochondria. Mitochondrial CRC was measured in isolated normal rat brain cortex (**A**) and cerebellum (**B**) mitochondria fluorometrically using succinate as a substrate, as described in Materials and Methods. Mitochondria were incubated for 2 min in the absence/presence of sodium pentobarbital at various concentrations (0.4–2 mM); then, pulses of Ca^2+^ (1.67 µM) were added until opening of mPTP. *—statistically significant effect, if compared to control, *p* < 0.05. Means ± SE of 6 separate experiments are presented.

In a further series of experiments, we tested whether sodium pentobarbital and ketamine exert a direct effect on CRC, respiration, and complex I activity in isolated normal cortical and cerebellar mitochondria. Figure 8 represents direct effect of sodium pentobarbital on CRC of mitochondria isolated from rat brain cortex (A) and cerebellum (B). As demonstrated in Figure 8A, 1.2 mM pentobarbital increased the CRC of cortex mitochondria from 50 ± 3 in control to 70 ± 7 nmol/mg Ca^2+^. Lower (0.4–0.8 mM) as well as higher concentrations (1.6–2 mM) of pentobarbital did not change the CRC in isolated cortex mitochondria. In contrast, sodium pentobarbital directly added to isolated cerebellum mitochondria at concentrations of 0.4–2 mM had no statistically significant effect on sensitivity of mitochondria to mPTP opening, measured as CRC (Figure 8B). Ketamine additions (0.2–1.0 mM) to isolated cortex and cerebellum mitochondria had no significant effect on CRC.

**Figure 9:**
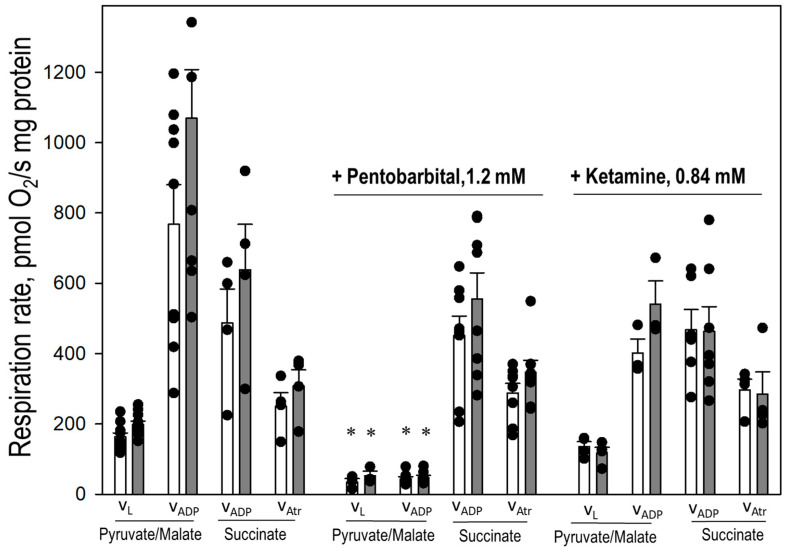
Direct effects of pentobarbital and ketamine on respiration of isolated cortex and cerebellum mitochondria. Mitochondrial oxygen consumption was measured as described in Materials and Methods. Mitochondrial LEAK respiration (V_L_) was measured using 1 mM pyruvate plus 1 mM malate) or 5 mM succinate plus 1.5 mM amytal. Mitochondrial ADP-stimulated respiration (V_ADP_) rate was achieved by adding 2 mM ADP. V_Atr_ was registered for succinate plus amytal oxidation with 100 μM atractyloside. For measurements of V_L_, V_ADP_, and V_Atr_, isolated mitochondria were incubated for 2 min in the presence of 1.2 mM of pentobarbital or 0.84 mM ketamine. *—statistically significant effect, if compared to control, *p* < 0.05. Means ± SE of 4 separate experiments are presented.

Next, we measured direct effect of sodium pentobarbital and ketamine on respiration in isolated cortex and cerebellum mitochondria (Figure 9). Mitochondria were incubated for 2 min in the presence of 1.2 mM of pentobarbital or 0.84 mM of ketamine, as well as the initial respiration rate (V_L_) and ADP-stimulated respiration rate (V_ADP_) with substrates pyruvate and malate; then, ADP-stimulated respiration rate and atractyloside-inhibited respiration (V_Atr_) with succinate were measured. As shown in Figure 9, 1.2 mM of pentobarbital added to suspension of isolated mitochondria induced inhibition of V_L_ and V_ADP_ in both cortex and cerebellum mitochondria, oxidizing pyruvate/malate: V_L_ was decreased from 162 and 193 to 26 and 40 pmol/O_2_/mg protein, in cortex and cerebellum, respectively; V_ADP_ was inhibited from 754 and 1055 to 43 and 45 pmol/O_2_/mg protein in cortex and cerebellum mitochondria, respectively. In contrast, V_ADP_ and V_Atr_ of mitochondria respiring with succinate of in the presence of sodium pentobarbital was not affected in either cortex or cerebellum mitochondria (Figure 9). The results indicate that sodium pentobarbital induces inhibition of V_L_ and V_ADP_ respiration in isolated brain cortex and cerebellum mitochondria respiring on complex I substrates pyruvate and malate, while oxidation of complex II substrate succinate was not affected. In addition, ketamine at concentration 0.84 mM did not significantly change V_L_ and ADP-stimulated respiration with substrates pyruvate/malate and succinate in isolated cortex and cerebellum mitochondria (Figure 9).

**Figure 10:**
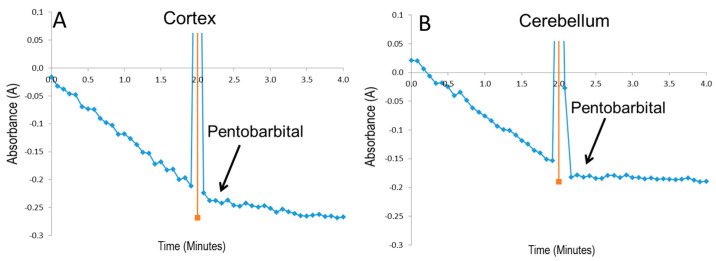
Direct effect of pentobarbital on complex I activity in freeze–thaw/sonicated cortex (**A**) and cerebellum (**B**) mitochondria. Representative original trace of measurements of complex I activity and effect of sodium pentobarbital on cortex (**A**) and cerebellum (**B**) mitochondria. Complex I activity was determined as reduced nicotinamide adenine dinucleotide (NADH) oxidation rate in frozen–thawed/sonicated mitochondria as described in Materials and Methods, *n* = 4.

Finally, we determined the direct effect of pentobarbital on complex I activity in cortical and cerebellar mitochondria disrupted by freeze–thaw/sonication. Pentobarbital (8 mM) strongly inhibited complex I activity by 84% in cortex and by 91% in cerebellum mitochondria, compared to complex I activity of non-treated control mitochondria (Figure 10A,B).

Altogether, these results indicate that millimolar concentrations of sodium pentobarbital exert direct effects on isolated brain mitochondria: pentobarbital decreases sensitivity of mPTP to Ca^2+^, inhibits pyruvate/malate oxidation and complex I activity. Ketamine (0.4–1.0 mM) did not exert any direct effect on CRC and respiration of isolated control cortex and cerebellum mitochondria.

## 4. Discussion

In the present study, we have demonstrated that anesthesia with pentobarbital/ketamine is protective against ischemia-induced rat brain cortex and cerebellum mitochondrial dysfunction and cell death, and that protective mechanism may be associated with the increased resistance of mitochondria to Ca^2+^-induced mPTP opening and improvement of mitochondrial respiratory function. We also showed that sodium pentobarbital but not ketamine can directly increase sensitivity of isolated cortical mitochondria to Ca^2+^-induced opening of mPTP and inhibits respiration with pyruvate/malate as well as complex I activity in isolated cortical and cerebellar mitochondria. These findings suggest that inhibition of mPTP opening may play an important role in the neuroprotective effects of barbiturates, which may have potential clinical value against cerebral ischemia/reperfusion injury.

The results of our study are in the agreement with data of other studies showing that inhibition of mitochondrial respiratory chain complex I by pyridaben protected neonatal mice brains against oxidative mitochondrial injury, reduced cerebral infarct volume, and restored mitochondrial Ca^2+^ buffering capacity following hypoxia/ischemia [20]. It has been also shown that inhibitor of complex I rotenone was an effective mPTP blocker in the liver, heart, and several cell lines [18,38]. Both pyridaben and rotenone are commonly used as pesticides [25] and are highly toxic to midbrain organotypic slices and neuroblastoma cells [39] and therefore cannot be potential therapeutic agents for patients with cerebral ischemia/hypoxia. In contrast, pentobarbital and other barbiturates, which are widely used in surgical anesthesia and treatment of sleep disorders and seizures, have been investigated extensively in many laboratories in human and animal models [40,41,42], though molecular mechanisms mediating protection against ischemic brain injuries were not completely understood. Protective effects of barbiturates have been suggested to be associated with their impact on reduction of cerebral metabolism; however, it has been shown that the neuroprotective effect of pentobarbital was unrelated to regulation of cerebral glucose utilization in a rat model of focal cerebral ischemia [43]. It has also been demonstrated that pentobarbital may inhibit the peroxidation of mitochondrial phospholipids during anoxia in animal brains [44] and in neonates with hypoxic ischemic encephalopathy [29]. In addition, in rat heart, amobarbital preserved mitochondrial functions and decreased mitochondrial ROS generation after ischemia/reperfusion [36]. In our study, we did not find any changes to ROS production in mitochondria isolated after ischemia with/without PBK anesthesia, indicating that mPTP was not modulated via ROS production, at least under our experimental conditions.

The mechanism of how complex I is linked to mPTP inhibition is under discussion [45]. Previously, it has been suggested that mPTP can be modulated by electron flow through complex I in isolated rat skeletal muscle mitochondria [46]. The authors found that mPTP opening was inhibited when mitochondrial respiration was partially inhibited and did not depend on differences in proton electrochemical gradient, Ca^2+^ uptake rates, or production of H_2_O_2_. Another study with rat brain mitochondria has demonstrated that stress-delayed mPTP opening were correlated, along with a mild inhibition of complex I and reduced O_2_ consumption in an ADP-stimulated state and uncoupled state, using complex I substrates [47]. It was found that complex I can exist in two different states, catalytically active and inactive; the transition to the inactive state may occur under pathological conditions, such as oxygen deprivation; and the physiological role of the inactive state of the complex I under ischemia/reperfusion is likely to protect mitochondria from ROS formation [48]. In our study, anesthesia with PBK caused partial inhibition of ADP stimulation and leak respiration in cortex and cerebellum mitochondria with pyruvate/malate and did not affect ROS generation. In parallel, pentobarbital added directly to isolated normal cortex and cerebellum mitochondria was able to inhibit pyruvate/malate respiration in both leak state and ADP-stimulated state, and almost fully blocked complex I activity. Succinate respiration in cortex and cerebellum mitochondria of PBK anesthesia control as well as when directly supplemented with pentobarbital was unaffected in both ADP-stimulated and leak states, therefore suggesting that complex II was not affected and is probably not related to mPTP regulation by pentobarbital. Importantly, recent evidence suggests that sensitivity of mPTP opening may depend on substrate availability and that upon mPTP opening, the respiratory rate of NADH-linked substrates was decreased, while FADH_2_-driven mitochondrial respiration remained at an elevated plateau [49].

In our study, ketamine, which also is a non-competitive N-methyl-D-aspartate (NMDA) receptor antagonist [50], was used in combination with pentobarbital to produce short- to medium-term surgical anesthesia. The literature displays conflicting views regarding the neurotoxic or neuroprotective effects of ketamine. It has been demonstrated that anesthesia with ketamine (90 mg/kg) reduced neuronal cell loss in the cortex after cerebral ischemia by improving the ratio of oxygen supply to consumption [51] or by suppressing sympathetic discharge [52]. In contrast, ketamine (40 mg/kg) in 7-day-old rat brains caused extensive neuronal cell death in the forebrain [53]. It has recently been reported that ketamine (50, 100, or 150 mg/kg IP) after 6 h treatment caused a mild inhibition in complex I activity and increased mitochondrial H_2_O_2_ generation [54]. However, in this study, ketamine did not change oxygen consumption in state 3 and increased oxygen consumption in state 4 when isolated mitochondria were energized with the complex I substrate. Other authors have shown that knockout mice with deficient mitochondrial complex I function showed increased resistance to ketamine [55]. Contrarily, sub-anesthetic doses of ketamine, 25 mg/kg led to significant increase in activities of mitochondrial complexes I, II, I-III, and IV in the prefrontal cortex, striatum, and hippocampus [56]. The actions of ketamine on the modulation of mitochondrial functions during brain ischemia are mostly unknown. Our results did not show any changes in the oxygen consumption or CRC of isolated control cortex and cerebellum mitochondria exposed to 0.2–1.0 mM ketamine. The results may vary due to the different duration of ketamine anesthesia, which can last up to 6 h [53] compared to the 20 min duration of PBK anesthesia in our study, whereas the direct effects of ketamine on CRC and respiration were measured after 2 min incubation with ketamine. On the other hand, ketamine toxicity may be modulated through NMDA-dependent pathways. Therefore, we suppose that pentobarbital/ketamine anesthesia-induced modulation of the mPTP and mitochondrial respiration during brain ischemia is rather mediated by pentobarbital inhibition of complex I.

In our study, the administration of PBK exerted an inhibitory effect on V_ADP_ with complex I substrates pyruvate/malate, which was higher with cortex mitochondria. In parallel, PBK before ischemia ameliorated succinate-dependent respiration in both cortex and cerebellum mitochondria, and pyruvate/malate-driven V_ADP_ in the cortex only. The overall trend showed that the protective effect of PBK after ischemia measured as V_ADP_ and cell necrosis level was more pronounced in the cortex compared to cerebellum. Moreover, the sodium pentobarbital was directly protective and increased the resistance to Ca^2+^-induced mPTP opening in isolated cortex mitochondria, not in cerebellum mitochondria. That the mPTP was more sensitive to Ca^2+^ in cortex mitochondria, whereas mitochondria from the cerebellum had lower sensitivity is in line with other studies, and this correlated with the susceptibility of these brain regions to ischemic damage [57]. However, we did not observe any differences in the effect of PBK on CRC and complex I activity in cortex and cerebellum mitochondria under ischemic conditions. Previously, we found that rotenone exerted similar protective effects on brain cell viability after ischemia in rat brain cortex and cerebellum, possibly via blockage of ischemia-induced opening of mPTP [21]. There are few data on what causes the differences in ischemia injury in the different brain regions, but it is thought it may correlate due to different ratios of neurons and non-neuron cells [58] and different sensitivities to Ca^2+^-induced mPTP [7,9].

## 5. Conclusions

In summary, the current study demonstrates that anesthesia with pentobarbital/ketamine prevents ischemia-induced death of rat brain cells, possibly due to a delay in Ca^2+^-induced mPTP opening and partial improvement in mitochondrial respiration function, and that respiratory chain complex I plays a key role in mPTP regulation. The present findings also elucidate a possible role of pentobarbital in modulation of mPTP opening, providing new data on a relevant clinical mechanism.

## Figures and Tables

**Table 1:** Effect of PBK on ROS production of cortex and cerebellum mitochondria.

	Control	120 min I	PBK	PBK + 120 min I
***Cortex***				
*Pyruvate/malate*	31.24 ± 5.12	44.63 ± 3.49	50.69 ± 5.05	49.92 ± 5.02
*Succinate*	33.60 ± 6.92	45.52 ± 4.21	49.65 ± 0.88	42.45 ± 4.81
***Cerebellum***				
*Pyruvate/malate*	27.77 ± 4.15	35.40 ± 2.68	32.98 ± 0.96	35.68 ± 2.03
*Succinate*	39.93 ± 5.92	51.30 ± 4.35	39.88 ± 4.99	37.50 ± 8.17

H_2_O_2_ generation by isolated cortex and cerebellum mitochondria was determined as described in Materials and Methods.

## Data Availability

The data presented in this study are available on request from the corresponding author. The data are not publicly available due to privacy restrictions.
